# Supplementary material for: Metallo-sideromycin as a dual functional complex for combating antimicrobial resistance
Source: Nat Commun. 2023 Sep 1;14:5311. doi: 10.1038/s41467-023-40828-3 (PMC10474269; doi:10.1038/s41467-023-40828-3)
Supplement: Supplementary file 1 — Supplementary Information [file 41467_2023_40828_MOESM1_ESM.pdf]

Supplementary Information

Metallo-sideromycin as a dual functional complex for combating antimicrobial resistance

Chenyuan Wang[1,5], Yushan Xia[1,5], Runming Wang[1], Jingru Li[1], Chun-Lung Chan[1], Richard Yi-Tsun Kao[2], Patrick H. Toy[1], Pak-Leung Ho[2,3], Hongyan Li[1,4]✉ and Hongzhe Sun[1,4]✉

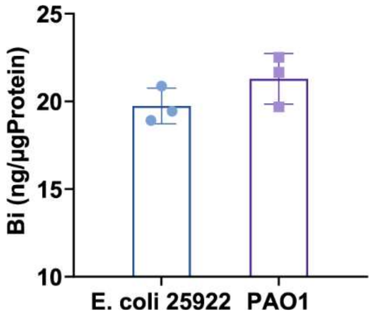

Supplementary Fig. 1 | Bismuth concentration per protein in cell lysates in the presence of CEF determined by ICP-MS. n=3 biologically independent samples. Error bars indicates mean ±SEM.

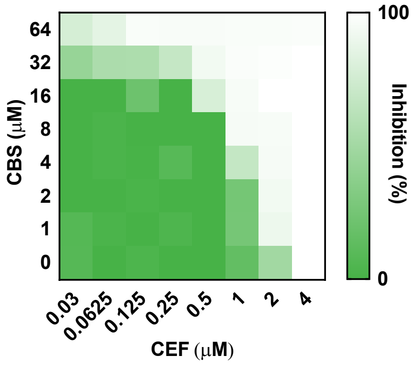

Supplementary Fig. 2 | Representative heat plot of microdilution checkerboard assay for the combination of CEF with CBS under iron deficient condition (M9 minimum medium) against PAO1. The FICI was calculated to be 0.24, indicative of synergy between them.

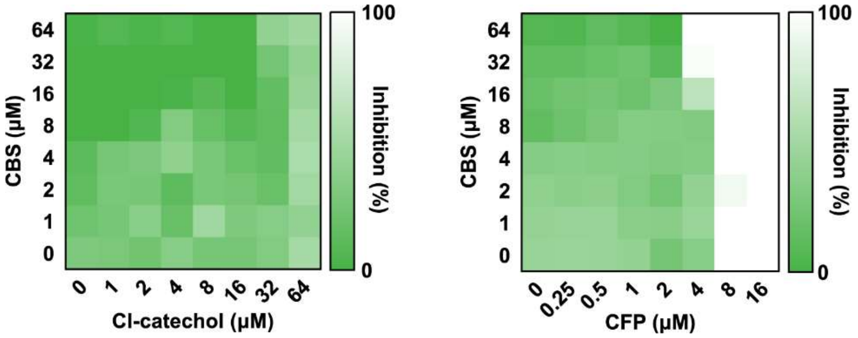

Supplementary Fig. 3 | Representative heat plot of microdilution checkerboard assay for the combination of 3-chlorocatechol with CBS and ceftazidime with CBS against PAO1. LB broth was used as a culture medium. The FICI was calculated to be 0.625.

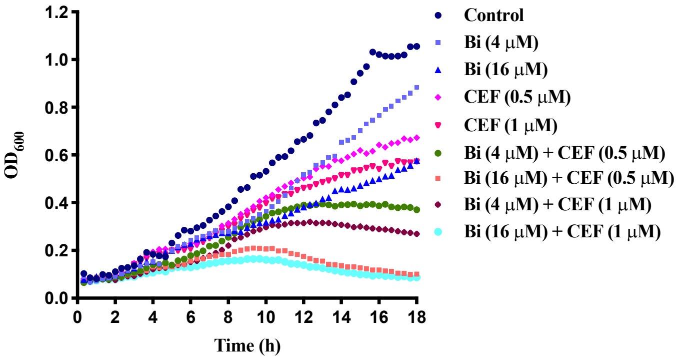

Supplementary Fig. 4 | Growth curves of PAO1 exposed to different concentration of CBS and CEF and their combination.

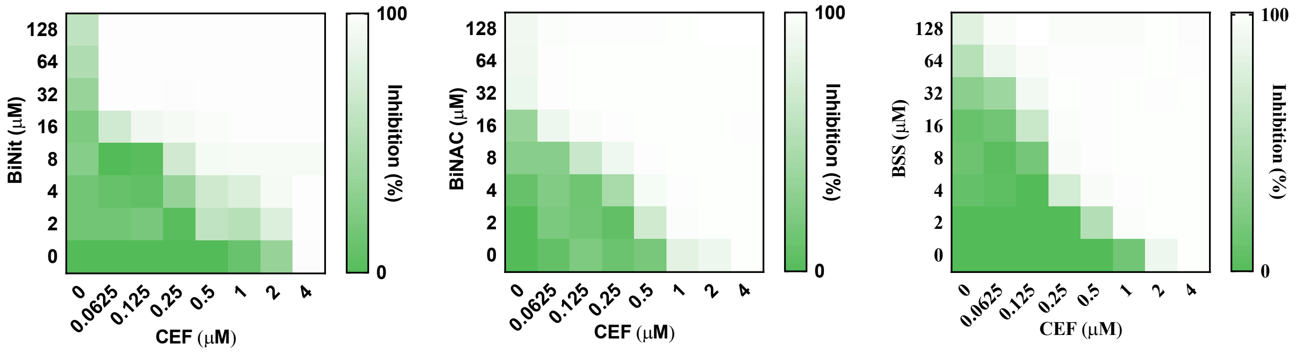

Supplementary Fig. 5 | Representative heat plots of microdilution checkerboard assay for the combination of CEF with different bismuth compounds including bismuth nitrate (BiNit), bismuth N-acetyl cysteine complex ($Bi(NAC)_3$), and bismuth subsalicylate (BSS) against PAO1. The FICIs were calculated to be 0.16, 0.28 and 0.14 (synergy is defined as FICI<0.5), respectively.

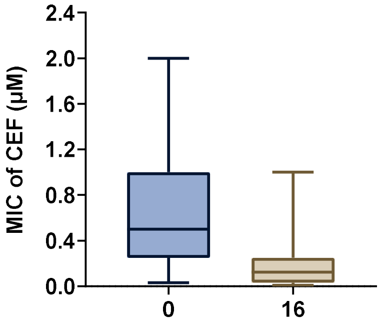

Supplementary Fig. 6 | Box plot showing the MIC of CEF against clinical *P.aeruginosa* strains with or without CBS. The concentration of CBS is 16 μM. Those strains with MICs more than 2-fold changes were considered as synergy and included in this figure. n= 45 clinical samples. The line in the middle of the box is plotted at the median (50th percentile), while the box extends from the 25th to 75th percentiles. The whiskers down to the minimum and up to the maximum values.

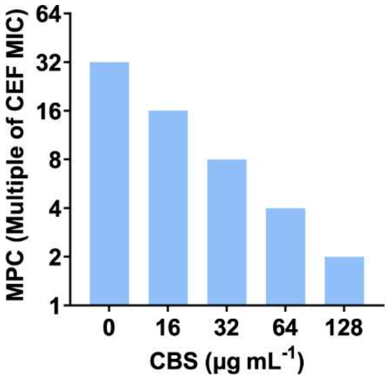

Supplementary Fig. 7 | MPC (Mutant Prevention Concentration) values of cefiderocol against PAO1 strain in the presence of different concentration of CBS ranging from 0 to 128 μg mL^{-1}.

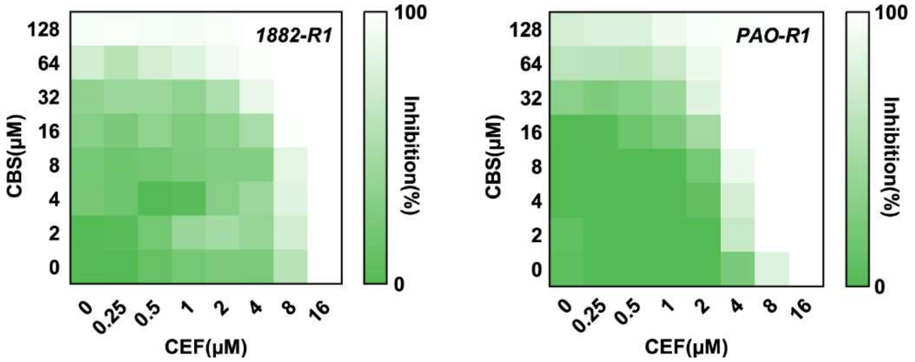

Supplementary Fig. 8 | Representative heat plots of microdilution checkboard assay for the combination of CEF and CBS against *1882-R1* and *PAO1-R1* strains.

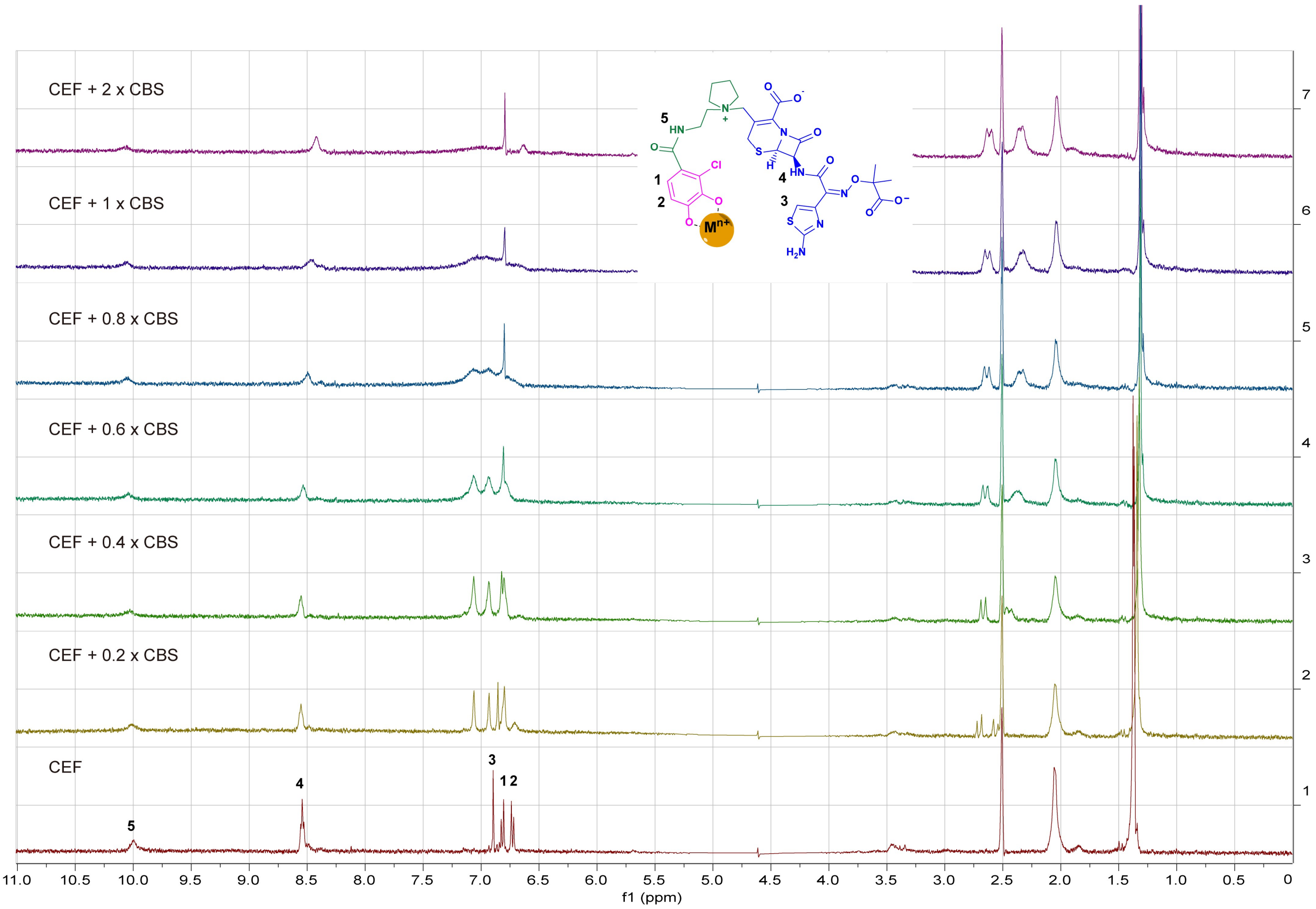

Supplementary Fig. 9 (a) ^{1}H NMR spectra of CEF in the presence of different molar ratio of CBS (0-2 molar equiv.) in mixed H_2O (90%) and DMSO-d6. The two doublets at 6.82 and 6.73 were assigned as the protons in 3-chlorocatechol (labeled as 1# and 2#). Note that addition of CBS resulted in decreased intensities and disappearance of these peaks, in the meantime, new peaks at 7.05 and 6.93 appeared and broadened with further addition of CBS, owing to the exchange between the free and bound forms of CEF on the ^{1}H NMR time scale.

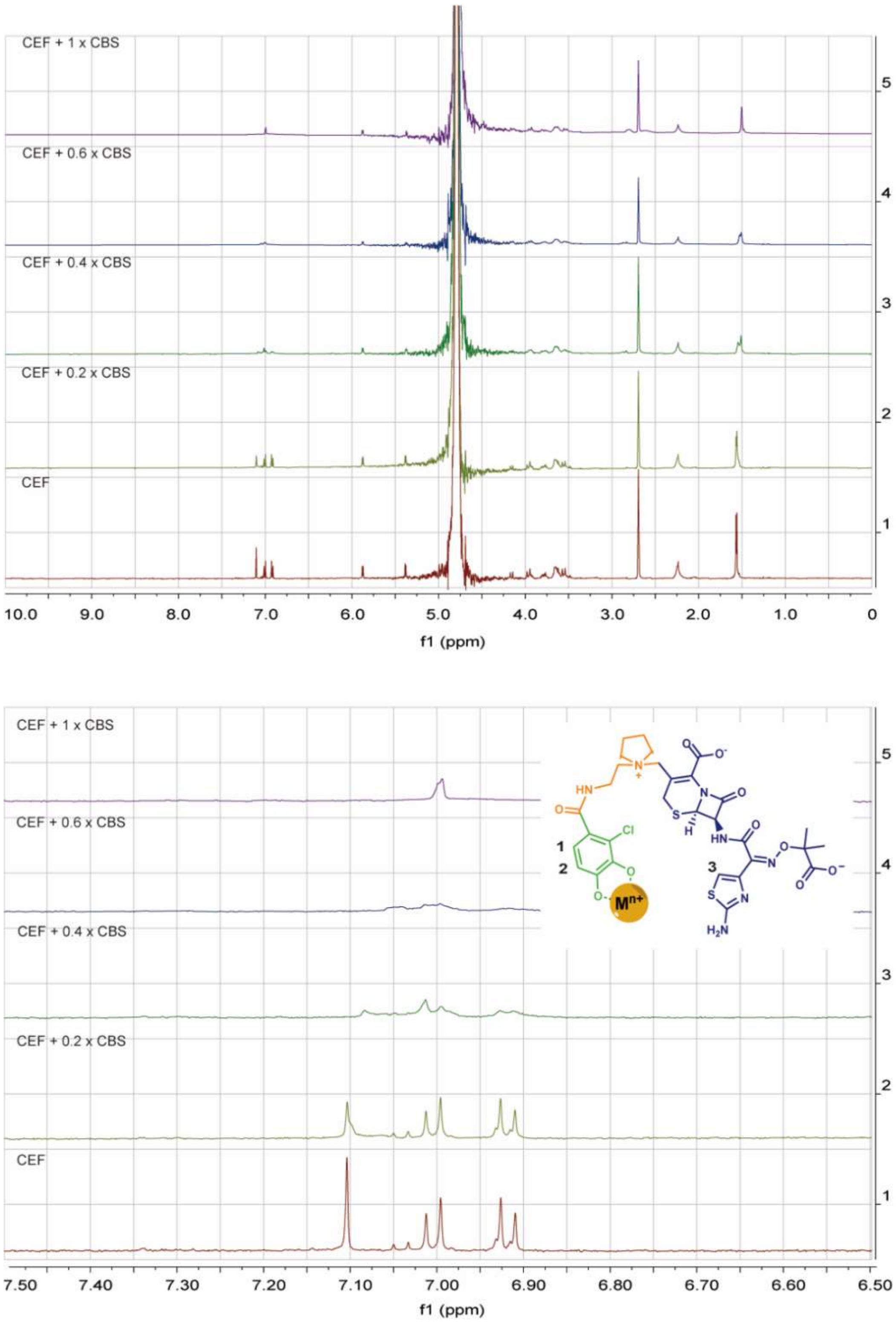

Supplementary Fig. 9 (b) ^{1}H NMR spectra of CEF in the presence of different molar ratio of CBS (0-2 molar equiv.) in mixed D_2O and DMSO-d6.

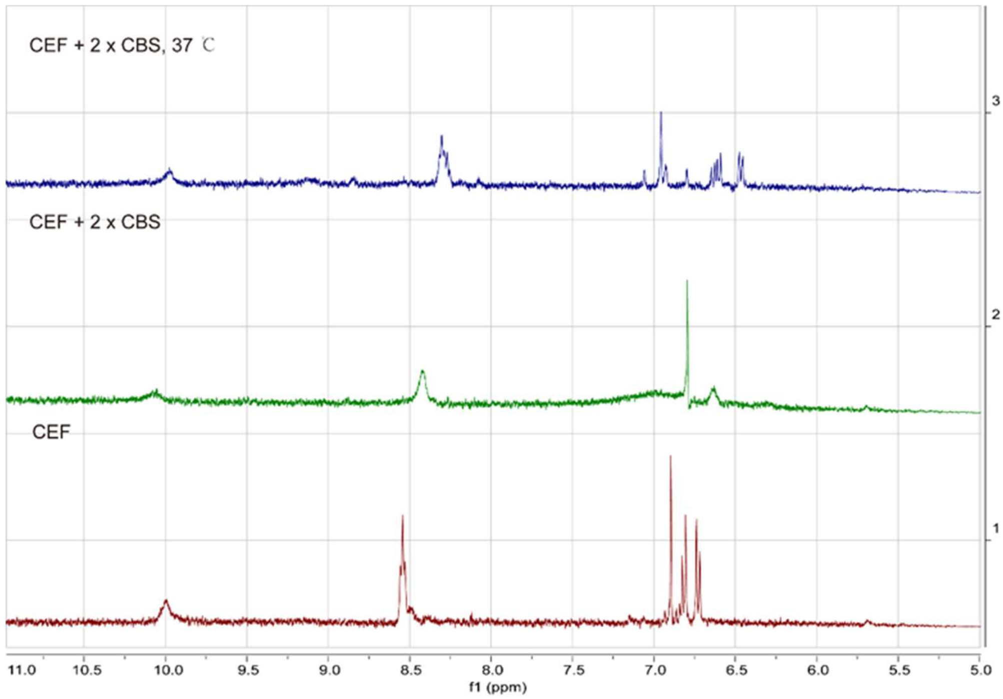

Supplementary Fig. 9 (c) ^{1}H NMR spectra of CEF with or without CBS in mixed H_2O and DMSO-d6 under room temperature and 37 ºC, respectively.

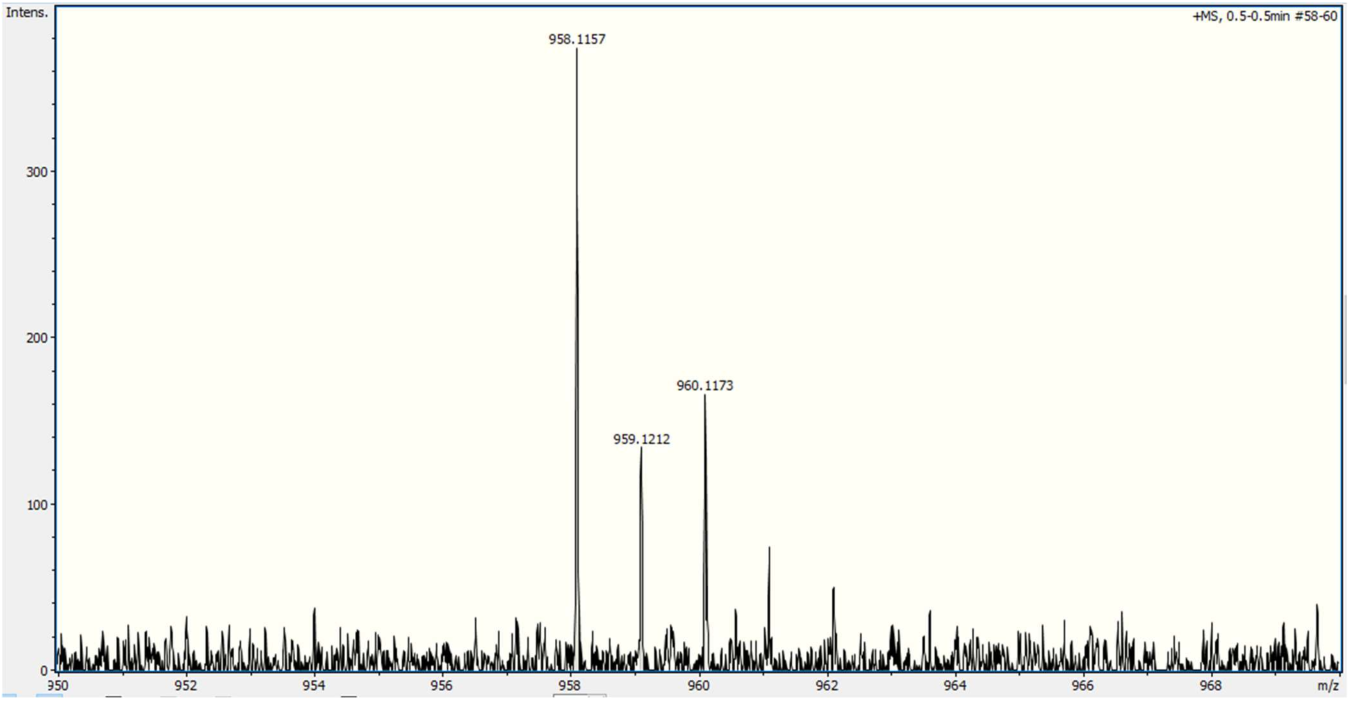

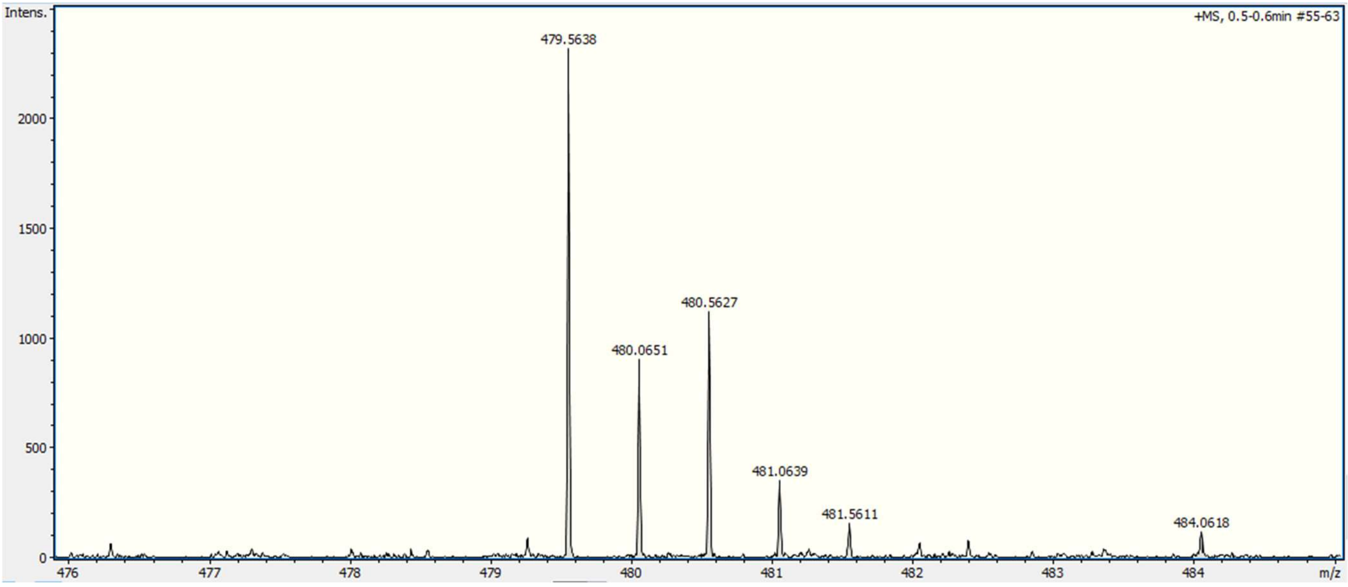

Supplementary Fig. 10 | MS spectra of bismuth(III)-CEF complex. Top: New peaks at m/z of 958.1157 assigned as $(Bi\text{-}CEF+H)^{+}$ (Cald. 958.1139). Bottom: 479.5638 as $(Bi\text{-}CEF+2H)^{2+}$ (Cald. 479.5605).

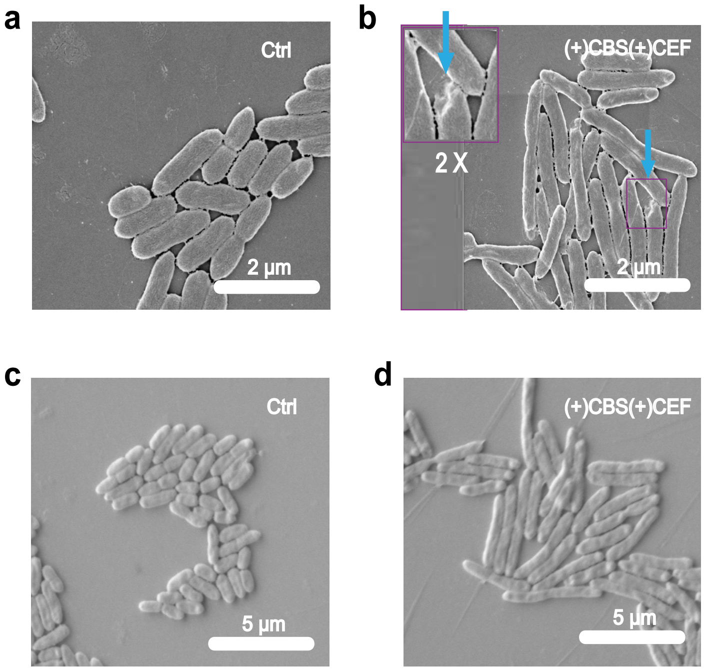

Supplementary Fig. 11 | SEM images of PAO1 cells without (a,c) or with (b,d) treatment of CBS and CEF combination for 30 min. Scale bar: 2 µm for (a, b) and 5 µm for (c, d). b and c are brightfired mode for providing depth information. Bacterial membrane disruption is marked with blue arrow and the two-fold blow-up is shown in the insert. Experiments were performed in triplicate.

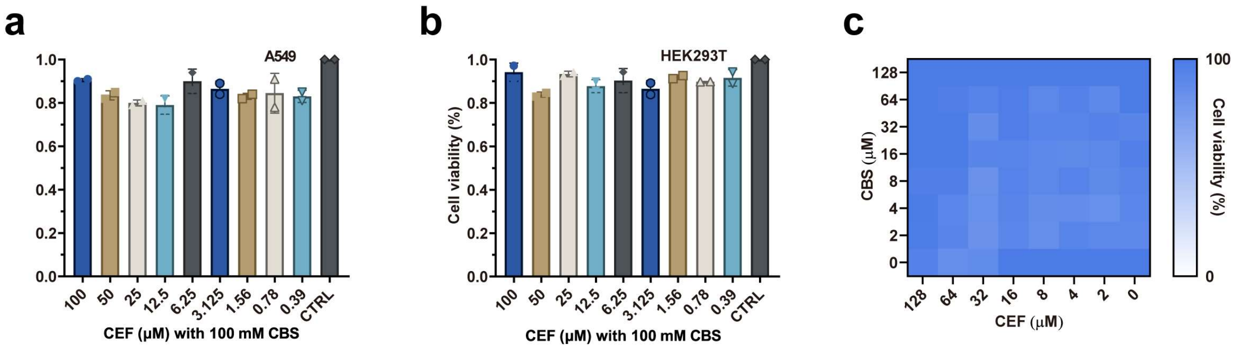

Supplementary Fig. 12 | Mammalian cell toxicity of bismuth-CEF combination. The cytotoxicity of combinatory CBS with CEF was evaluated through examination of the viability of the human lung tissue cell A549 and the embryonic kidney cells HEK293T by MTT assay. **a,** The viability of A549 cell treated with different concentrations of CEF and 100 µM CBS. n=2 biologically independent samples. Mean value of two replicates are shown and error bars indicates ±SEM. **b,** The viability of HEK293T cell treated with different concentrations of CEF and 100 µM CBS. **c,** Representative heat plot of cell viability for the combination of CBS and CEF at varying concentrations. n=2 biologically independent samples. Mean value of two replicates are shown and error bars indicates ±SEM.

Table S1 | Antibacterial activity of CEF in the absence and presence with different metal compounds against different bacteria

	MIC of CEF (μM)							
		In the presence of 50 μM of metal compounds						
**Strains**	**Alone**	**Bi^{3+}**	**Ga^{3+}**	**Co^{3+}**	**Cr^{3+}**	**Fe^{3+}**	**Mn^{2+}**	**Ti^{4+}**
A. baumannii ATCC-AYE	16	16	16	16	16	>16	16	16
A. baumannii clinical 27107	1	0.5	1	4	1	4	1	2
K. pneumoniae ATCC7003	16	16	16	16	16	16	16	16
K. pneumoniae clinical 24063	0.25	0.125	0.5	1	0.25	2	0.5	0.25
K. pneumoniae (KPC-2)[+]	8	8	16	16	8	>16	8	>16
E. aerogenes (MCR-1)[+]	16	16	16	16	16	>16	16	16
Salmonella enterica	0.06	0.25	0.06	0.25	0.06	0.25	0.06	0.06
Aeromonas hydrophila	1	0.5	1	4	1	4	1	2
Vibrio cholerae	0.5	0.5	0.5	1	0.5	1	0.5	0.25
Proteus mirabills	8	8	8	8	8	16	16	8

Metal compounds used are: Colloidal bismuth citrate (CBS), $Ga(NO_3)_3$, $Co(OAc)_3$, $Cr_2(SO_4)_3$, $FeCl_3$, $Mn(OAc)_2$, Ti(IV)-citrate.

Table S2 | Antibacterial activity of CEF in the absence and presence with CBS against clinical *P. asruginosa* strains

	**MIC of CEF (μM)**			
**CBS (mg/L)**	**0**	**4**	**8**	**16**
**Strains**				
2110	0.5	0.5	0.5	0.25
2111	1	1	1	1
2112	0.125	0.25	0.25	0.25
2113	2	1	1	1
2114	0.5	0.25	0.125	0.125
2115	1	0.5	0.5	0.5
2116	0.5	0.5	0.5	0.5
2117	0.25	0.25	0.125	0.125
2118	0.5	0.5	0.25	0.125
2119	2	1	0.5	0.5
2120	1	1	1	1
2121	1	1	1	1
2122	0.5	0.25	0.25	0.0625
2123	2	0.5	0.25	0.25
2124	2	2	2	2
2125	0.25	0.25	0.25	0.25
2126	2	2	2	2
2127	0.5	0.25	0.25	0.25
2128	1	1	1	1
2129	1	0.5	0.5	0.5
2130	0.5	0.5	0.5	0.25
2131	0.5	0.5	0.5	0.25
2132	0.5	0.125	0.031	0.016
2133	0.5	0.0625	0.016	0.008
2134	0.25	0.125	0.063	0.016
2135	0.25	0.25	0.25	0.125
2136	1	0.125	0.063	0.031
2137	0.031	0.008	0.008	0.008
2138	0.25	0.063	0.016	0.008
2139	1	0.5	0.5	0.25
2140	0.25	0.016	0.008	0.008

	MIC of CEF (μM)			
**CBS (mg/L)**	**0**	**4**	**8**	**16**
**Strains**				
2030	0.5	0.5	0.25	0.25
2031	0.5	0.5	0.25	0.25
2032	0.5	1	2	2
2033	1	1	1	1
2034	0.5	0.25	0.25	0.25
2035	2	2	2	2
2036	2	1	1	1
2037	1	0.5	0.25	0.0625
2038	1	1	1	1
2039	0.25	0.125	0.0625	0.0625
2040	0.25	0.25	0.125	0.125
2041	2	0.5	0.25	0.125
2042	1	1	0.5	0.5
2043	1	1	0.5	0.5
2044	1	0.5	0.125	0.0625
2045	0.25	0.125	0.0625	0.031
2046	0.125	0.031	0.031	0.015
2047	0.5	0.5	0.25	0.25
2048	0.031	0.008	0.008	0.008
2049	0.5	0.125	0.0625	0.031
2050	2	0.25	0.125	0.0625
2051	0.5	0.25	0.125	0.0625
2052	0.25	0.25	0.125	0.0625
2053	0.5	1	1	1
2054	2	2	2	1
2055	1	1	1	1
2056	0.5	0.5	0.5	0.5
2057	0.5	0.125	0.031	0.031
2058	2	1	1	0.5
2059	1	1	1	1
2060	0.25	0.25	0.25	0.25

Table S3 | Bacterial strains, plasmids and primers used in this study

Strain/Plasmid/Primer	Description	Source (Reference)
P. aeruginosa		
PAO1	Wild type strains of *pseudomonas aeruginosa*	Lab collection
PA14	Wild type strains of *pseudomonas aeruginosa*	Lab collection
ATCC27853	Wild type strains of *pseudomonas aeruginosa*	Lab collection
PAO1*ΔpiuA*	PAO1 deleted of *piuA*	This study
PAO1-GFP	PAO1 with pEX18Tc-gfp	This study
PA1882	Clinical isolate	HKU medicine collected
Clinical-R1	Serial passages cultured PA1882 after 12 passages	This study
Clinical-R2	Serial passages cultured PA1882 after 12 passages	This study
2110-2140, 2030-2060	Clinical isolates	HKU medicine collected
PAO1-R1	Serial passages cultured PAO1 after 12 passages	This study
PAO1-R2	Serial passages cultured PAO1 after 12 passages	This study
PA245	Clinical isolates, meropenem, ceftazidime, piperacillin-lazobatam, cefepime, ciprofloxacin, cefiderocol resistant	HKU medicine collected
PA247	Clinical isolates, meropenem, ceftazidime, piperacillin-lazobatam, cefepime, ciprofloxacin, cefiderocol resistant	HKU medicine collected
**Plasmids**		
pEX18Tc	Gene replacement vector, TET^r, $oriT^+$, $sacB^+$	Lab collection
pEX18Tc-*ΔpiuA*	*piuA* gene of PAO1 deletion on pEX18Tc	This study
**Primer**	**Sequence (5' – 3')**	**Function**
pEX18Tc-R	ACCTGCAGGCAUGCAAGCTTG	Gene deletion
pEX18Tc-F	ACTCTAGAGGAUCCCCGGGTAC	Gene deletion
piuA-F-UF	ATGCCTGCAGGUTTGTGGTAGGTACCGGTGAGG	*piuA* deletion; RT-PCR
piuA-F-UR	AAGTGGAAGTUAGAAACGGCGGUATCCGTG	*piuA* deletion
piuA-R-UF	AACTTCCACTUCTGACTGCCG	*piuA* deletion
piuA-R-UR	ATCCTCTAGAGUCTGGCGGATGCTGTTCCATAC	*piuA* deletion; RT-PCR

Other bacterial strains mentioned in the primary screening are all lab collected strains.

Table S4 | Raw data of ICP-MS

Sample					209 Bi [No Gas Mode]	
Rjct	Data File	Acq. Date-Time	Type	Sample Name	CPS	CPS RSD
FALSE	W1.d	8/2/2019 2:25 PM	Sample	W1	3826.423333	3.8676813
FALSE	W2.d	8/2/2019 2:27 PM	Sample	W2	3115.143333	5.5306526
FALSE	W3.d	8/2/2019 2:28 PM	Sample	W3	2629.876667	3.4157782
FALSE	S1.d	8/2/2019 2:31 PM	Sample	S1	536957.07	2.1414819
FALSE	S2.d	8/2/2019 2:33 PM	Sample	S2	616551.22	0.868548
FALSE	S3.d	8/2/2019 2:35 PM	Sample	S3	810427.7767	1.0475871
FALSE	S4.d	8/2/2019 2:37 PM	Sample	S4	539172.4267	3.2330148
FALSE	S5.d	8/2/2019 2:39 PM	Sample	S5	1271773.567	0.946548
FALSE	S6.d	8/2/2019 2:41 PM	Sample	S6	4845255.607	2.0738973
FALSE	S7.d	8/2/2019 2:43 PM	Sample	S7	722538.9333	1.0276201
FALSE	S8.d	8/2/2019 2:45 PM	Sample	S8	9999941.427	0.9237292
FALSE	S9.d	8/2/2019 2:47 PM	Sample	S9	23457252.15	1.413838
FALSE	S10.d	8/2/2019 2:49 PM	Sample	S10	81529427.75	3.2755631
FALSE	S11.d	8/2/2019 2:50 PM	Sample	S11	86137268.59	5.276999
FALSE	S12.d	8/2/2019 2:52 PM	Sample	S12	201935571.2	3.6335057
FALSE	1.d	8/2/2019 2:54 PM	Sample	1	130270.6767	2.8152441
FALSE	2.d	8/2/2019 2:56 PM	Sample	2	54847.36	2.3289752
FALSE	3.d	8/2/2019 2:58 PM	Sample	3	14340.17333	3.4747293
FALSE	4.d	8/2/2019 3:00 PM	Sample	4	8202.246667	1.897346
FALSE	5.d	8/2/2019 3:01 PM	Sample	5	16865116.81	4.5616067
FALSE	6.d	8/2/2019 3:26 PM	Sample	6	4683747.127	1.7549764
FALSE	7.d	8/2/2019 3:28 PM	Sample	7	4912540.967	3.4601299
FALSE	8.d	8/2/2019 3:31 PM	Sample	8	324636729.5	2.8610378
FALSE	9.d	8/2/2019 3:33 PM	Sample	9	328753137	4.3296365
FALSE	10.d	8/2/2019 3:36 PM	Sample	10	230470827.4	3.1570612
FALSE	11.d	8/2/2019 3:38 PM	Sample	11	231117516.3	3.6287211
FALSE	12.d	8/2/2019 3:40 PM	Sample	12	408162457.3	4.5666638
FALSE	13.d	8/2/2019 3:42 PM	Sample	13	432947628.2	0.2327409
FALSE	14.d	8/2/2019 3:45 PM	Sample	14	1285068.517	7.9673177
FALSE	15.d	8/2/2019 3:47 PM	Sample	15	1150274.23	2.2349462
FALSE	16.d	8/2/2019 3:49 PM	Sample	16	2514488.003	1.5384519
FALSE	17.d	8/2/2019 3:51 PM	Sample	17	2703052.49	5.8996899
FALSE	18.d	8/2/2019 3:53 PM	Sample	18	240656501.7	0.6087814
FALSE	19.d	8/2/2019 3:54 PM	Sample	19	240655199.8	2.4853715
FALSE	20.d	8/2/2019 3:56 PM	Sample	20	363732485.9	2.6835586
FALSE	21.d	8/2/2019 3:58 PM	Sample	21	351104389.3	3.5747844
FALSE	22.d	8/2/2019 4:00 PM	Sample	22	345958522.6	1.0106486
FALSE	23.d	8/2/2019 4:02 PM	Sample	23	336206633.4	2.3473359
FALSE	24.d	8/2/2019 4:04 PM	Sample	24	2559502.43	2.1664822
FALSE	25.d	8/2/2019 4:06 PM	Sample	25	1874302.82	2.0062267
FALSE	26.d	8/2/2019 4:08 PM	Sample	26	2859953.19	2.0156787
FALSE	27.d	8/2/2019 4:10 PM	Sample	27	3145753.573	5.4482671
FALSE	28.d	8/2/2019 4:12 PM	Sample	28	327329511	4.6100445
FALSE	29.d	8/2/2019 4:14 PM	Sample	29	318793010.8	2.5513359
FALSE	30.d	8/2/2019 4:16 PM	Sample	30	455452850.9	2.0044671
FALSE	31.d	8/2/2019 4:18 PM	Sample	31	463344428.9	5.474385

FALSE	32.d	8/2/2019 4:20 PM	Sample	32	473225514.3	0.5185972
FALSE	33.d	8/2/2019 4:22 PM	Sample	33	465192854.8	3.1085909
FALSE	34.d	8/2/2019 4:24 PM	Sample	34	9120978.097	1.6988441
FALSE	35.d	8/2/2019 4:26 PM	Sample	35	8216317.893	2.6564827
FALSE	36.d	8/2/2019 4:27 PM	Sample	36	12700276.33	5.761217
FALSE	37.d	8/2/2019 4:29 PM	Sample	37	12604280.95	2.3368612
FALSE	38.d	8/2/2019 4:31 PM	Sample	38	2333298852	6.7755923
FALSE	39.d	8/2/2019 4:33 PM	Sample	39	2437495017	2.6463014
FALSE	40.d	8/2/2019 4:34 PM	Sample	40	430265780	2.0960122
FALSE	41.d	8/2/2019 4:36 PM	Sample	41	415049535.2	2.0034872
FALSE	42.d	8/2/2019 4:38 PM	Sample	42	177589489.2	7.0765278
FALSE	43.d	8/2/2019 4:40 PM	Sample	43	173494472.4	2.6349525
FALSE	44.d	8/2/2019 4:41 PM	Sample	44	5392872.227	1.864683
FALSE	45.d	8/2/2019 4:43 PM	Sample	45	4898304.217	0.7845558
FALSE	46.d	8/2/2019 4:45 PM	Sample	46	18969867.09	1.4578635
FALSE	47.d	8/2/2019 4:46 PM	Sample	47	18576138.26	2.8346475
FALSE	48.d	8/2/2019 4:48 PM	Sample	48	343316548.4	5.5385077
FALSE	49.d	8/2/2019 4:50 PM	Sample	49	331411170.4	2.6132748
FALSE	50.d	8/2/2019 4:51 PM	Sample	50	288284686.1	2.7741833
FALSE	51.d	8/2/2019 4:53 PM	Sample	51	275574713.6	2.411003
FALSE	52.d	8/2/2019 4:55 PM	Sample	52	130767969.6	2.1005303
FALSE	53.d	8/2/2019 4:57 PM	Sample	53	133187702.1	4.9718161
FALSE	54.d	8/2/2019 4:59 PM	Sample	54	493271.33	2.9101766
FALSE	55.d	8/2/2019 5:01 PM	Sample	55	361578.0533	0.8955759
FALSE	56.d	8/2/2019 5:03 PM	Sample	56	105365.96	1.9896177
FALSE	57.d	8/2/2019 5:04 PM	Sample	57	104919.1	0.6009478
FALSE	58.d	8/2/2019 5:06 PM	Sample	58	265545587.4	4.3861499
FALSE	59.d	8/2/2019 5:08 PM	Sample	59	269075267.1	3.4084592
FALSE	60.d	8/2/2019 5:09 PM	Sample	60	163701411.1	7.1997728
FALSE	61.d	8/2/2019 5:11 PM	Sample	61	154689425.7	15.923232
FALSE	62.d	8/2/2019 5:13 PM	Sample	62	1025746593	6.9103037
FALSE	63.d	8/2/2019 5:15 PM	Sample	63	1016835660	2.5364121
FALSE	64.d	8/2/2019 5:16 PM	Sample	64	3176058.58	2.1467498
FALSE	65.d	8/2/2019 5:18 PM	Sample	65	2665599.537	1.7173724
FALSE	66.d	8/2/2019 5:20 PM	Sample	66	391141.7333	0.9004951
FALSE	67.d	8/2/2019 5:22 PM	Sample	67	370800.65	2.8055505
FALSE	68.d	8/2/2019 5:23 PM	Sample	68	169451622.3	4.2253812
FALSE	69.d	8/2/2019 5:25 PM	Sample	69	175687246.5	1.1723341
FALSE	70.d	8/2/2019 5:27 PM	Sample	70	121387182.3	1.4376731
FALSE	71.d	8/2/2019 5:29 PM	Sample	71	128482273.2	0.6600736
FALSE	72.d	8/2/2019 5:30 PM	Sample	72	70294901.57	2.1599898
FALSE	73.d	8/2/2019 5:32 PM	Sample	73	70559611.3	3.4132027
FALSE	74.d	8/2/2019 5:33 PM	Sample	74	6908122.377	2.6443564
FALSE	75.d	8/2/2019 5:35 PM	Sample	75	6567678.503	3.7355703
FALSE	76.d	8/2/2019 5:37 PM	Sample	76	89468.46667	3.6610166
FALSE	77.d	8/2/2019 5:39 PM	Sample	77	106859.1833	0.8803354
FALSE	78.d	8/2/2019 5:40 PM	Sample	78	62823152.79	5.9694936
FALSE	79.d	8/2/2019 5:42 PM	Sample	79	60750774.97	2.7824103
FALSE	80.d	8/2/2019 5:44 PM	Sample	80	79979504.57	5.1809947
FALSE	81.d	8/2/2019 5:45 PM	Sample	81	82073089.8	4.6190778

FALSE	82.d	8/2/2019 5:47 PM	Sample	82	43163772.72	5.129606
FALSE	83.d	8/2/2019 5:49 PM	Sample	83	40978268.51	2.0122406
FALSE	84.d	8/2/2019 5:50 PM	Sample	84	2978042.283	1.0377902
FALSE	85.d	8/2/2019 5:52 PM	Sample	85	2614559.46	1.4049379
FALSE	86.d	8/2/2019 5:54 PM	Sample	86	90631.08333	4.2730615
FALSE	87.d	8/2/2019 5:56 PM	Sample	87	89371.18333	1.3931904
FALSE	88.d	8/2/2019 5:57 PM	Sample	88	106290389.4	2.1489235
FALSE	89.d	8/2/2019 5:59 PM	Sample	89	114053432.2	3.9323945
FALSE	90.d	8/2/2019 6:01 PM	Sample	90	104881590.3	5.9779762
FALSE	91.d	8/2/2019 6:03 PM	Sample	91	97940328.12	3.214776
FALSE	92.d	8/2/2019 6:04 PM	Sample	92	53102749.8	5.2077853
FALSE	93.d	8/2/2019 6:06 PM	Sample	93	50328749.27	1.2502446
FALSE	94.d	8/2/2019 6:08 PM	Sample	94	886937.61	0.2807565
FALSE	95.d	8/2/2019 6:09 PM	Sample	95	773397.5133	2.4390828
FALSE	96.d	8/2/2019 6:11 PM	Sample	96	435183.3633	2.0748703
FALSE	97.d	8/2/2019 6:13 PM	Sample	97	431027.4233	1.1244603
FALSE	98.d	8/2/2019 6:15 PM	Sample	98	51195307.16	2.290516
FALSE	99.d	8/2/2019 6:17 PM	Sample	99	54744122.98	4.5740188
FALSE	100.d	8/2/2019 6:18 PM	Sample	100	109433661.7	3.7724735
FALSE	101.d	8/2/2019 6:20 PM	Sample	101	106214359.8	1.7765801
FALSE	102.d	8/2/2019 6:22 PM	Sample	102	55766517.91	4.229977
FALSE	103.d	8/2/2019 6:23 PM	Sample	103	55016159.56	5.0949296
FALSE	104.d	8/2/2019 6:25 PM	Sample	104	793359.6467	1.9883876
FALSE	105.d	8/2/2019 6:27 PM	Sample	105	390366.1933	1.4659339
FALSE	106.d	8/2/2019 6:29 PM	Sample	106	470373.6667	0.4997784
FALSE	107.d	8/2/2019 6:30 PM	Sample	107	462025.3033	1.5736925
FALSE	108.d	8/2/2019 6:32 PM	Sample	108	35417920.47	2.2953565
FALSE	109.d	8/2/2019 6:34 PM	Sample	109	36293103.36	1.1113767
FALSE	120.d	8/2/2019 6:36 PM	Sample	120	90304413.13	3.2282641
FALSE	121.d	8/2/2019 6:37 PM	Sample	121	91047504.81	4.7414388
FALSE	122.d	8/2/2019 6:39 PM	Sample	122	34758417.67	3.1844647
FALSE	123.d	8/2/2019 6:41 PM	Sample	123	33070494.72	6.93542
FALSE	124.d	8/2/2019 6:42 PM	Sample	124	387915.6067	1.7314265
FALSE	125.d	8/2/2019 6:44 PM	Sample	125	290690.3567	3.1876996
FALSE	126.d	8/2/2019 6:46 PM	Sample	126	398017.8933	1.7714843
FALSE	127.d	8/2/2019 6:48 PM	Sample	127	386823.9933	1.1116273
FALSE	128.d	8/2/2019 6:49 PM	Sample	128	9406364.91	1.1622245
FALSE	129.d	8/2/2019 6:51 PM	Sample	129	9568110.053	3.9032258
FALSE	130.d	8/2/2019 6:53 PM	Sample	130	82648886.11	3.675007
FALSE	131.d	8/2/2019 6:54 PM	Sample	131	85225991.72	3.4399699
FALSE	132.d	8/2/2019 6:56 PM	Sample	132	44274247.28	7.3242785
FALSE	133.d	8/2/2019 6:58 PM	Sample	133	43446974.12	3.1779625
FALSE	134.d	8/2/2019 7:00 PM	Sample	134	209429.35	2.2839416
FALSE	135.d	8/2/2019 7:02 PM	Sample	135	184732.64	1.1281789
FALSE	136.d	8/2/2019 7:23 PM	Sample	136	137860.9233	4.1242127
FALSE	137.d	8/2/2019 7:24 PM	Sample	137	78995.09	8.1121683
FALSE	138.d	8/2/2019 7:25 PM	Sample	138	67864.18	2.0517017

*The bismuth level was further nomolized by protein concentration per sample.
