# Supplementary material for: Metallo-sideromycin as a dual functional complex for combating antimicrobial resistance
Source: Nat Commun. 2023 Sep 1;14:5311. doi: 10.1038/s41467-023-40828-3 (PMC10474269; doi:10.1038/s41467-023-40828-3)
Supplement: Supplementary file 3 — Reporting Summary [file 41467_2023_40828_MOESM3_ESM.pdf]

nature portfolio

Corresponding author(s): Hongyan Li, Hongzhe Sun

Last updated by author(s): Jul 17, 2023

Reporting Summary

Nature Portfolio wishes to improve the reproducibility of the work that we publish. This form provides structure for consistency and transparency in reporting. For further information on Nature Portfolio policies, see our Editorial Policies and the Editorial Policy Checklist.

Statistics

For all statistical analyses, confirm that the following items are present in the figure legend, table legend, main text, or Methods section.

n/a	Confirmed	
☐	☒	The exact sample size (*n*) for each experimental group/condition, given as a discrete number and unit of measurement
☐	☒	A statement on whether measurements were taken from distinct samples or whether the same sample was measured repeatedly
☐	☒	The statistical test(s) used AND whether they are one- or two-sided<br>*Only common tests should be described solely by name; describe more complex techniques in the Methods section.*
☐	☒	A description of all covariates tested
☐	☒	A description of any assumptions or corrections, such as tests of normality and adjustment for multiple comparisons
☐	☒	A full description of the statistical parameters including central tendency (e.g. means) or other basic estimates (e.g. regression coefficient) AND variation (e.g. standard deviation) or associated estimates of uncertainty (e.g. confidence intervals)
☐	☒	For null hypothesis testing, the test statistic (e.g. *F*, *t*, *r*) with confidence intervals, effect sizes, degrees of freedom and *P* value noted<br>*Give P values as exact values whenever suitable.*
☒	☐	For Bayesian analysis, information on the choice of priors and Markov chain Monte Carlo settings
☒	☐	For hierarchical and complex designs, identification of the appropriate level for tests and full reporting of outcomes
☒	☐	Estimates of effect sizes (e.g. Cohen's *d*, Pearson's *r*), indicating how they were calculated

Our web collection on statistics for biologists contains articles on many of the points above.

Software and code

Policy information about availability of computer code

Data collection	NMR was performed on a Bruker 500 MHz NMR spectrometer; SpectraMax iD3 was used for OD reading; ICP-MS was performed by Agilent 7500a, Agilent Technologies, CA, USA; UV-vis spectra were recorded on a Varian Cary 50 spectrophotometer; SEM imaging was performed by High Resolution Schottky FE-SEM, TESCAN MAIA3 XMN; Confocal laser scanning microscopy (Leica TCS SP8 CLSM) was used for Biofilm formation measurement
Data analysis	Prism 9.0 (GraphPad Software Inc.), MestReNova X64 14.3.3

For manuscripts utilizing custom algorithms or software that are central to the research but not yet described in published literature, software must be made available to editors and reviewers. We strongly encourage code deposition in a community repository (e.g. GitHub). See the Nature Portfolio guidelines for submitting code & software for further information.

Data

Policy information about availability of data

All manuscripts must include a data availability statement. This statement should provide the following information, where applicable:

- Accession codes, unique identifiers, or web links for publicly available datasets
- A description of any restrictions on data availability
- For clinical datasets or third party data, please ensure that the statement adheres to our policy

All data are available in the main text or the supplementary materials.

Research involving human participants, their data, or biological material

Policy information about studies with human participants or human data. See also policy information about sex, gender (identity/presentation), and sexual orientation and race, ethnicity and racism.

Reporting on sex and gender	NA
Reporting on race, ethnicity, or other socially relevant groupings	NA
Population characteristics	NA
Recruitment	NA
Ethics oversight	NA

Note that full information on the approval of the study protocol must also be provided in the manuscript.

Field-specific reporting

Please select the one below that is the best fit for your research. If you are not sure, read the appropriate sections before making your selection.

☒ Life sciences ☐ Behavioural & social sciences ☐ Ecological, evolutionary & environmental sciences

For a reference copy of the document with all sections, see nature.com/documents/nr-reporting-summary-flat.pdf

Life sciences study design

All studies must disclose on these points even when the disclosure is negative.

Sample size	No sample size calculation was performed
Data exclusions	No data were excluded
Replication	All experiments were performed in triplicate
Randomization	All experimental groups were allocated randomly
Blinding	Blinding was not relevant

Reporting for specific materials, systems and methods

We require information from authors about some types of materials, experimental systems and methods used in many studies. Here, indicate whether each material, system or method listed is relevant to your study. If you are not sure if a list item applies to your research, read the appropriate section before selecting a response.

Materials & experimental systems

n/a	Involved in the study	
☒	☐	Antibodies
☐	☒	Eukaryotic cell lines
☒	☐	Palaeontology and archaeology
☐	☒	Animals and other organisms
☒	☐	Clinical data
☒	☐	Dual use research of concern
☒	☐	Plants

Methods

n/a	Involved in the study	
☒	☐	ChIP-seq
☒	☐	Flow cytometry
☒	☐	MRI-based neuroimaging

Eukaryotic cell lines

Policy information about cell lines and Sex and Gender in Research

Cell line source(s)	HEK-293-T Cells and A549 cells were purchased from Sigma-Aldrich

Authentication	HEK-293-T Cell line and A549 cell line were authenticated by STR-PCR
Mycoplasma contamination	All cell lines tested negative for mycoplasma contamination
Commonly misidentified lines (See ICLAC register)	No misidentified cell lines were used.

Animals and other research organisms

Policy information about studies involving animals; ARRIVE guidelines recommended for reporting animal research, and Sex and Gender in Research

Laboratory animals	6-8 weeks-old, female BALB/c mice
Wild animals	No wild animals were used in the study
Reporting on sex	No sex-based analyses have been performed. Murine Acute Pneumonia Model of Pseudomonas aeruginosa lung infection was performed in female BALB/c mice based on the protocal.
Field-collected samples	No Field-collected samples were used in the study
Ethics oversight	All animal experiments were approved by and performed in accordance with the guidelines approved by Committee on the Use of Live Animals in Teaching and Research (CULATR), the University of Hong Kong.

Note that full information on the approval of the study protocol must also be provided in the manuscript.
